# Editorial for the Special Issue “Latest Review Papers in Molecular Oncology 2023”

**DOI:** 10.3390/ijms25063257

**Published:** 2024-03-13

**Authors:** Carmine Stolfi

**Affiliations:** Department of Systems Medicine, University of Rome “Tor Vergata”, 00133 Rome, Italy; carmine.stolfi@uniroma2.it

Human cancers are products of multistep processes resulting in abnormal cell growth and differentiation, along with a loss of apoptotic function, leading to the uncontrolled expansion of neoplastic cells and their spread to surrounding tissues and, ultimately, distant parts of the body [1]. Cancer can originate from any of the cells present in the body; thus, there are more than a hundred distinct types of cancer, which vary substantially in terms of behavior and response to therapy [1]. As such, cancer is one of the most common causes of death worldwide due to often late diagnosis and the lack of efficient therapeutic options for patients with advanced-stage diseases [2].

This Special Issue comprises nineteen review articles written by leading international expert in specific cancer research fields, touching on various aspects of cancer development and progression, as well as treatment options. It is not this Editorial’s objective to provide a detailed description of each of the works; rather, it will encourage the reader to explore them in depth.

Four of the published studies focused on understanding molecular and cellular events’ causal roles in tumors’ transformation from a benign to a malignant state and the cancer progression.

The modulation of endoplasmic reticulum stress (ERS) activation, a key hallmark of cancer that helps transformed cells cope with hypoxia, a lack of vascularization in early-stage tumors, and scarce nutritional conditions [3], is an interesting strategy for selective cancer treatment due to the different adaptive phenotypes of ERS that exist between tumor and normal cells [4,5]. Correia de Sousa et al. (contribution 1) reviewed and discussed the relevance of ERS activation or suppression for renal-cell carcinoma (RCC) and the therapeutic potential of targeting this cellular process.

Recent studies revealed that inflammasomes (defined as cytosolic multiprotein oligomers of the innate immune system responsible for activating inflammatory responses) contribute to the pathophysiology, development, and progression of myeloid malignancies [6]. The study by Parciante and colleagues (contribution 2) explored the role of the nucleotide-binding domain (NOD)-like receptor protein 3 (NLRP3), the most frequently studied inflammasome type, in myeloproliferative neoplasms, a set of uncommon neoplastic blood disorders in which the bone marrow produces pathological amounts of red blood cells, white blood cells, or platelets [7].

Immunotherapies based on immune checkpoint blockade have shown remarkable clinical outcomes and durable responses in patients with many tumor types [8]. The programmed death protein 1 (PD-1) is a common immunosuppressive member present on the surfaces of T cells crucial to the downregulation of the immune system and advancements in self-tolerance. Its ligand-programmed cell death ligand 1 (PD-L1) is overexpressed on the surfaces of malignant tumor cells, where it binds to PD-1, inhibits the proliferation of PD-1-positive cells, and enables the immune evasion of tumors, leading to treatment failure. Therefore, the PD-1/PD-L1-based pathway is vital for cancer immunotherapy and has become regarded an important immune checkpoint in recent years, as demonstrated by the fact that PD-1/PD-L1 inhibitors have demonstrated clinical efficacy in many tumors [9]. Kuszczak et al. (contribution 3) investigated the role of the PD-1/PD-L1 pathway and B-cell CLL/lymphoma 2 (BCL-2) family proteins, with the latter involved in intrinsic apoptosis pathway regulation, as well as myelodysplastic syndrome pathogenesis, a heterogeneous group of closely related clonal hematopoietic disorders common in elderly populations and characterized by one or more peripheral blood cytopenia [10]. Lastly, the authors focused on results derived from clinical trials of BCL-2 family protein inhibitors and PD-1/PD-L1 inhibitors.

Optimal cancer therapeutic approaches sometimes involve simultaneously targeting multiple molecules found in both the tumor and supportive tissues. Chalcones are plant-derived polyphenols that have received increased attention due to their multi-target anticancer activities, as well as because they allow the relatively easy structural manipulation and synthesis of new chalcone derivatives. The article by Michalkova et al. (contribution 4) summarized and discussed the experimental evidence on the anticancer effects of natural and synthetic chalcones, focusing on stomach cancer and colorectal cancer (CRC). In particular, the authors reported that the observed chemotherapeutic effects of chalcones were mediated by, among other things, the modulation of several signaling pathways (e.g., Wnt/β-catenin, nuclear factor kappa B, mitogen-activated protein kinase), reactive oxygen species induction, and angiogenesis inhibition.

The accumulated evidence indicates that the evolutionary mechanism of cancer is driven not only by mutations and genetic alterations but also by non-genetic, often non-heritable determinants, such as environmental challenges and cell plasticity [11]. The former (contributions 5 and 6) and latter (contributions 7 and 8) topics have both been covered by studies published in this Special Issue.

A higher urbanization rate, higher number of adult populations at risk due to exposure to certain lifestyles, and higher number of workers actively exposed to environmental carcinogens are significant cancer risk factors [12]. In particular, the increased incidence of brain tumors, observed more frequently in industrialized countries [13,14], has generated scholarly interest in investigating the roles of different pollutants in modulating the risk of brain tumorigenesis. Pagano’s work (contribution 5) described the association between environmental carcinogens and brain cancer risk, focusing on specific categories of pollutants (such as pesticides, heavy metals, nitrites, and fine microparticles) and their sources.

The increasing frequency of general and, in particular, male cancers, coupled with the reduction in male fertility seen worldwide, suggests a potential evolutionary link between these two phenomena. In this context, Erenpreisa and co-workers’ study (contribution 6) proposed that social stress and endocrine disruption caused by environmental pollution with organic materials, which alter sex determination in male fetuses and further spermatogenesis in adults, could increase the risk of developing cancer.

During neoplastic transformation’s earliest stages, epigenetic reprogramming causes genomic instability and subsequent transcriptome alteration, causing differentiated cells to re-acquire cell plasticity. In fact, it is well established that a critical component of cancer pathogenesis is the progressive loss of cellular identity and escape from a differentiated state [15].

In recent years, epigenome dysregulation associated with the loss of cell identity and functions has emerged as a crucial feature of glioblastoma pathogenesis, the most common and aggressive malignant primary brain tumor [16], and epigenetic therapy, which involves manipulating the cancer epigenome by targeting epigenetic factors, gaining increasing attention as a possible curative approach [17]. Gargano and colleagues (contribution 7) examined the advantages and disadvantages of strategies used to treat glioblastoma by targeting Bromodomain and Extra-Terminal Domain (BET) proteins, a family of multifunctional epigenetic readers, primarily involved in transcriptional regulation through chromatin modeling [18].

Finally, focusing on MCF-7 breast cancer cells as a system model, Tsuchiya et al. (contribution 8) provided an overview of the complex systems theory-based approaches used to investigate cell fate change’s genomic mechanism.

Despite recent advances in cancer treatment, cancer cells’ intrinsic and/or acquired resistance to therapies, the lack of efficacy of immunotherapeutics for many patients, and the side effects of current cytotoxic agents still represent major limitations [19]. These issues reinforce the need to better understanding cancer pathogenesis to discover and validate more effective and safe therapeutic options.

Six articles (contributions 9 to 14) considered different issues that cause drug resistance and/or tumor regrowth, as well as possible approaches to circumvent these issues.

For cancer therapy, plant-derived molecules and, in particular, flavonoids have exhibited good anti-neoplastic activity with minimal side effects, representing an effective, inexpensive, and accessible therapeutic approach [20]. Luiz-Ferreira’s study (contribution 9) applied compounds belonging to natural flavonoid families (such as flavones, flavonols, isoflavones, chalcones, and prenylflavonoids) to overcome cancer cells’ resistance to tumor necrosis factor-related apoptosis-inducing ligand (TRAIL)-induced programmed cell death, highlighted the underlying molecular mechanisms, and discussed potential future perspectives and drawbacks.

Based on the assumption that the microbiome, that is, the collection of all microbes (e.g., bacteria, fungi, viruses) that naturally live on our bodies and inside us, directly modulates the anticancer immune response both at the intestinal level and systemically [21], Sillo et al. (contribution 10) reviewed the available literature on the composition of the microbiome that occurs in DNA mismatch repair-deficient (microsatellite instability-high) and DNA mismatch repair-proficient (microsatellite instability-low) CRC, as well as its role in the response to immunotherapy.

Most solid malignancies comprise not only tumor cells but also various types of immune cells that exert both immunosuppressive functions (e.g., tumor-associated macrophages, regulatory T cells) and tumor-fighting functions (e.g., cytotoxic CD8+ T cells, natural killer cells), cancer-associated fibroblasts, endothelial cells, various additional tissue-resident cell types, and multiple extracellular soluble molecules (e.g., cytokines, growth factors, chemotactic factors) [22]. Increasing evidence shows that this complex and heterogeneous ecosystem, defined as the tumor microenvironment, affects all stages of cancer progression (from tumor initiation and progression to metastatic dissemination and outgrowth) and responses to therapies [23]. Habanjar’s article (contribution 11) comprehensive overviewed the crosstalk and biological roles of different cytokines (that is, leptin, IL-1β, IL-6, IL-8, IL-23, IL-17, IL-10, IL-2, IL-12, and IFN-γ) in the breast tumor microenvironment, recommending possible immunotherapeutic strategies.

Cancer cells use epigenetic changes to exhibit significant resistance to chemotherapeutic drugs. Therefore, targeting epigenetic modifications could be a promising strategy for overcoming drug resistance [24]. Oura et al. (contribution 12) described recent findings regarding the influence of epigenetic regulation and the tumor microenvironment on resistance to systemic therapy in hepatocellular carcinoma (HCC), the most common histological type, accounting for approximately 90% of all primary liver cancers [25], focusing on approaches to improve therapeutic outcomes for patients with advanced-stage disease.

Damage-associated molecular patterns (DAMPs), also called alarmins or danger signals, are endogenous molecules released from necrotic cells that die after exposure to various stressors [26]. After binding to their receptors, they stimulate various signaling pathways in target cells. DAMPs are especially abundant in malignant tumors’ microenvironments and have been proposed to affect the behaviors of malignant and stromal cells in multiple ways, often promoting cell proliferation, migration, invasion, and metastasis, as well as increased immune evasion. Zapletal et al. (contribution 13) initially summarized cell necrosis’ main characteristics and the diverse methods used to assess this type of cell death in clinical practice. Next, they discussed the importance of necrosis as a prognostic factor, concluding by focusing on DAMPs and their role in the tumor microenvironment.

Drug resistance is one of the main obstacles to the treatment of most patients with multiple myeloma (MM), the second most prevalent hematologic cancer after non-Hodgkin’s lymphoma and identified based on monoclonal plasma cell proliferation in bone marrow [27]. The study of Bashiri and Tabatabaeian (contribution 14) delved into the possibility of targeting autophagy, a cellular process through which cells break down and recycle cellular components, including damaged proteins and organelles, to overcome drug resistance and improve the treatment effectiveness in MM cells. 

Four research studies published in this Special Issue reviewed and discussed current (contributions 15 and 16) and up-and-coming (contributions 17 and 18) clinical approaches for improving responses to therapy and outcomes in cancer patients.

The metastasis of cancer to the central nervous system (CNS) and acquired resistance complicate the treatment of advanced non-small cell lung cancer (NSCLC) involving driver gene alterations, such as anaplastic lymphoma kinase (ALK) rearrangements [28]. Therefore, treatment strategies that focus on brain metastases are essential for managing patients with this neoplasia. Ando and colleagues (contribution 15) performed a network meta-analysis of relevant phase III randomized controlled trials to compare the efficacies of different ALK inhibitors (crizotinib, ceritinib, alectinib, brigatinib, antacatinib, and lorlatinib) used to treat patients with ALK rearrangement-positive advanced NSCLC. In particular, data derived from generation-specific comparisons showed that the third-generation ALK inhibitor lorlatinib was significantly more effective than second-generation ALK inhibitors, first-generation ALK inhibitors, and chemotherapy at prolonging the progression-free survival of the subgroup of patients with CNS metastases.

Chemotherapeutic drugs’ effectiveness can be limited by their rapid metabolism, their toxic side effects, and the development of cancer cell resistance [29]. To overcome these drawbacks, various delivery strategies employing liposomes—small artificial spherical vesicles created from cholesterol and natural non-toxic phospholipids [30]—have been investigated over the years. Indeed, liposomes can improve the stability and biodistribution of cytotoxic drugs, overcome tissue and cellular uptake limitations at target sites in vivo, and reduce systemic adverse effects associated with non-encapsulated agents. It is noteworthy that liposomal formulations of various chemotherapeutic drugs (for example, doxorubicin and irinotecan) have received regulatory approval and are routinely used in clinics. The study by Fulton and Najahi-Missaoui (contribution 16) provided a comprehensive overview of the main properties of liposomes and the current statuses of liposome-mediated cancer therapies, as well as limitations and challenges related to achieving maximal therapeutic efficacy.

Renal cell carcinoma (RCC) represents approximately 3% of all adult malignancies, and advanced metastatic disease correlates with a poor survival rate. [2]. Among all histologic variants, clear cell RCC (ccRCC) is the most common, representing 70% to 90% of all renal carcinomas, whereas non-clear cell renal cell carcinomas (ncRCC) are a heterogeneous group subclassified into different subtypes with genetic and biochemical characteristics that differentiate them from each other and ccRCC [31]. Negative clinical outcomes in patients with RCC result from the demanding management of the disease from diagnosis to treatment and follow-up (for example, for small renal masses and cystic lesions, the differential diagnosis of benign or malignant tissues has potential pitfalls when imaging or even renal biopsy is applied), worsened by the low efficiency of currently available therapeutic approaches in the metastatic stage, in particular with respect to the ncRCC subtypes that, due to their rarity and poor molecular characterization, are managed using systemic, untailored, and rarely curative treatments [32]. Recent advances in artificial intelligence, imaging techniques, and genomics may help clinicians to determine the stratification risk, treatment selection, the follow-up strategy, and the prognosis of RCC. In this context, the combination of radiomics (the method of extracting a large number of features from medical images using data characterization algorithms) and radiogenomics, which entails a correlation between quantitative or qualitative imaging features and the genomic data obtained from tissue analysis, as well as other clinical data, has achieved good results and could individualize tumor therapy and treatment instruments. Ferro and colleagues (contribution 17) summarized and discussed current advantages and disadvantages, limitations, and future perspectives regarding these state-of-the-art techniques to allow for tailored and more effective management of patients with RCC. 

Finally, the paper written by Blanco et al. (contribution 18) addressed the possibility of using local intratumor delivery of monoclonal antibodies (mAbs) to reduce the risk of developing serious toxicities while maintaining therapeutic efficacy. Though this goal can be achieved through several strategies, the most attractive means is the use of gene therapy vectors expressing mAbs within the tumor mass. The authors outlined the basic, translational, and clinical results of intratumor mAb delivery, together with descriptions of non-viral and viral strategies for mAb delivery in preclinical and clinical development.

As mentioned above, cancer is a complex and multifaceted disease. Indeed, a tumor can be considered as a growing and evolving system that undergoes multiple changes to become cancerous, resist antitumor agents, and induce inter- and intratumoral cellular and genetic variability, making it a unique disease for every patient. In addition, cancer cells actively modify neighboring normal cells, forming tumor microenvironments, and evolve together [33]. Obviously, this heterogeneity makes the effective use of molecularly targeted medicine extremely unfavorable. The hypothesis-generating article written by Alekseenko et al. (contribution 19) highlighted some general principles regarding the organization and evolution of cancerous tumors. The authors singled out two supramolecular characteristics common to all tumors: the clustered nature of tumor interactions with their microenvironment and the formation of biomolecular condensates (that is, membraneless compartments) with tumor-specific distinctive features, proposing that these features, once tested and validated in further studies, could form the basis of strategies for employing tumor-specific supramolecular targeted therapies.

In conclusion, the review articles published in this Special Issue span different fields of cancer research, making substantial contributions to our understanding of cancer’s global disease burden and providing a basis for further translational investigations, particularly for patients who do not adequately benefit from currently available therapies. Finally, I wish to thank the authors for their valuable contributions and the reviewers and editors for their excellent support. I hope that the articles included in this Special Issue meet the reader’s expectations and further promote cancer treatment research among the scientific community.

## Data Availability

Not applicable.
